# Factors Influencing Peri-Extraction Anxiety: A Cross-Sectional Study

**DOI:** 10.3390/dj12060187

**Published:** 2024-06-20

**Authors:** Wojciech Niemczyk, Agnieszka Balicz, Karolina Lau, Tadeusz Morawiec, Janusz Kasperczyk

**Affiliations:** 1 Department of Dental Surgery, Faculty of Medical Sciences in Zabrze, Medical University of Silesia, 40-055 Katowice, Poland; abalicz@sum.edu.pl (A.B.); tmorawiec@sum.edu.pl (T.M.); 2 Department of Environmental Medicine and Epidemiology, Faculty of Medical Sciences in Zabrze, Medical University of Silesia, 40-055 Katowice, Poland; karolina.lau@sum.edu.pl (K.L.); jkasperczyk@sum.edu.pl (J.K.)

**Keywords:** dental anxiety, tooth extraction, dentistry, oral surgery

## Abstract

Background: Fear and anxiety are common psychological responses to unpleasant stimuli, with dental fear being the fourth most prevalent type of fear or phobia. However, not all dental procedures cause the same level of anxiety, with dental surgery and tooth extraction being among the top five most frightening procedures in the field. Tooth extractions are also the most common surgical procedure in dental surgery. It is important to manage anxiety in the dental office by identifying the main factors. Methods: The study was conducted on a group of 250 patients. The survey technique and the Beliefs about Pain Control Questionnaire (BPCQ) were used in the study. Pain and stress intensity on a Visual Analog Scale (VAS) were measured in each patient before and after tooth extraction procedures. Results: Young women and people from small towns had the highest levels of anxiety. The factor causing the highest level of anxiety was fear of complications. Patients undergoing extraction of retained teeth were characterized by higher levels of anxiety. Conclusions: Perioperative stress is strongly dependent on numerous factors. For patient comfort, it may be crucial for dentists to have knowledge about these factors and the ability to utilize them to reduce stress before and after tooth extractions.

## 1. Introduction

Dental anxiety is a universal phenomenon that affects individuals of all ages across diverse geographical locations. The condition has a negative impact on the oral health-related quality of life of children and adults alike [1]. Anxiety associated with the thought of visiting the dentist for preventive care and other dental procedures is referred to as dental anxiety. It was cited as the fifth-most common cause of anxiety by Agras et al. [2]. Approximately 25% of the population experiences dental anxiety, with multiple individuals meeting the criteria for dental anxiety [3]. However, not all dental procedures cause the same level of anxiety. Dental surgery, particularly having a tooth extracted, is known to be among the top five most frightening procedures in dental practice [4,5]. It is also one of the most common procedures in the field of oral surgery [6,7,8,9]. Tooth extraction is rarely life-threatening and has a relatively short recovery period. Nevertheless, the physical and psychological impact makes it a stressful experience [10]. This may be caused by the fact that many factors contribute to the heightened anxiety. Such factors are fear of experiencing pain or discomfort during the extraction procedure, the perceived loss of control, and the uncertainty surrounding the outcome of the treatment [11]. In a multifactorial aetiology model, Wide et al. [12] demonstrated that previous negative dental experiences (e.g., painful experiences in the dental field) play a fundamental role in the development of dental anxiety. In a clinical sample of Italian dental patients, Scandurra et al. found that the likelihood of having high dental anxiety was greater with more exposure to traumatic dental events [13]. Dental anxiety is a complex phenomenon caused by several variables. Pain is often cited as both an aetiological and a maintaining factor in patients’ dental anxiety [10]. Walburn et al. in their meta-analysis showed that stress is closely related to wound healing in patients [14]. Marucha et al. also showed such a relationship in patients with oral mucosal wounds [15]. The psycho-emotional status of patients undergoing various dental procedures is also very important for patient–doctor comfort during the procedure [16]. Therefore, it is evident that managing anxiety and fear in the dental office is important. To achieve this, it is crucial to identify the factors causing fear of the dentist using established scales for measuring anxiety [4]. The study’s authors aimed to determine the factors affecting anxiety before and immediately after tooth extraction, and how perioperative anxiety is influenced by a patient’s pain beliefs.

## 2. Materials and Methods

### 2.1. Study Design

This single-centre study was conducted in a cross-sectional design using a questionnaire as the study tool. Therefore, the study was conducted and reported in adherence with the guidelines for reporting cross-sectional studies, in which a questionnaire was used as the study tool [17,18,19].

### 2.2. Data Collection

The study was conducted using a questionnaire consisting of 31 questions divided into 4 sections. The Section 1 was basic data such as gender, age, and education level. The Section 2 dealt with pre-surgical factors affecting anxiety. The Section 3 was a standardized Beliefs about Pain Control Questionnaire (BPCQ) [20] consisting of 13 questions. The Section 4 of the questionnaire was conducted after the surgery and concerned the evaluation of both individual intra- and postoperative factors. The 10-point Visual and Analogue Scale (VAS) [21] was used to measure levels of stress and pain.

### 2.3. Cohort Characteristics

The 250 respondents included 150 women and 100 men within an age range of 11–84 years and a mean age of 29.41 ± 12.82. Of the total patients, 200 had undergone extraction of retained teeth, and the remaining 50 had extraction of fully erupted teeth. Because of the single-center nature of the study, the patient base had limited variation.

All 250 respondents were admitted to an academic dental centre in Bytom, Poland. The inclusion and exclusion criteria for patient qualification are presented in Table 1.

**Figure 1:**
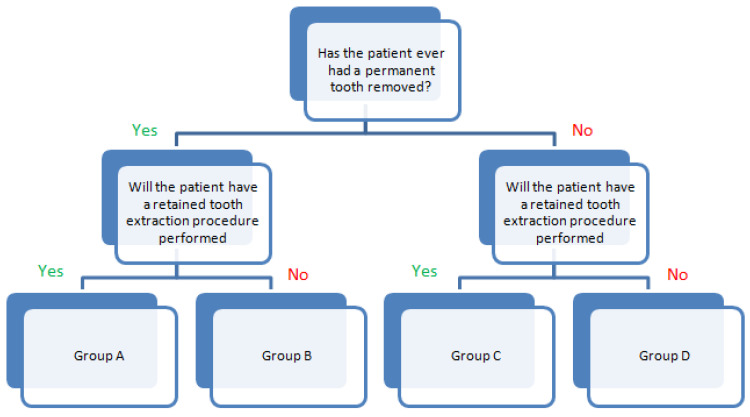
Distribution of respondents into different groups. Group A—patients who have previously undergone tooth extraction and who are now referred to a dental clinic for the removal of a retained tooth. Group B—patients who have previously undergone tooth extraction and who are now referred to a dental clinic for the removal of an erupted tooth. Group C—patients who have not previously undergone tooth extraction and who are now referred to a dental clinic for the removal of a retained tooth. Group D—patients who have not previously undergone tooth extraction and who are now referred to a dental clinic for the removal of an erupted tooth.

The patients were assigned to one of four groups according to their extraction history, pain complaints, level of tooth eruption, and whether they had been referred by their treating physician for oral sanitation. Figure 1 illustrates the process of assigning patients to the most appropriate study group in order to ensure transparency of the results.

### 2.4. Survey Administration

A simple random sampling technique was employed during data collection. The survey was conducted by two surgeons between 1 March and 15 April 2023. Both survey and extraction staff collected responses from respondents in the same predetermined manner. The initial section of the survey, pertaining to preoperative considerations, was collected during the dental procedure itself. Conversely, the subsequent section, addressing postoperative concerns, was filled immediately following the extraction and the subsequent administration of post-extraction instructions. Following each of these stages, the survey was evaluated to ascertain that all questions had been answered. All surveys were completed in their original format and then manually entered into an electronic database. In order to minimise the potential for human error in data entry, each survey was subject to double-checking by separate individuals.

### 2.5. Study Preparation

Prior to the commencement of data collection, the two clinicians established the appropriate stages of the visit, during which patients were required to respond to a series of questions. Prior to tooth extraction, patients were asked questions related to preoperative stress and the BPCQ. Questions related to the visit were not administered until after extraction and post-extraction recommendations were provided.

### 2.6. Statistical Analysis

The data were subjected to a thorough examination for completeness and accuracy, after which they were entered into a database created in MS Excel 16.70. The data were then analysed using the Statistica 13.0 package. Intergroup comparisons were conducted for unrelated quantitative variables, after testing assumptions (Shapiro–Wilk test, Levene’s test), using parametric tests (*t*-tests). When assumptions were not met, non-parametric tests (Whitney’s U–Mann test) were employed. For qualitative variables, tests were used. Pearson’s Chi-square and the highest reliability were employed. In all analyses, a value of *p* < 0.05 was considered to be statistically significant.

## 3. Results

### 3.1. Participants

**Figure 2:**
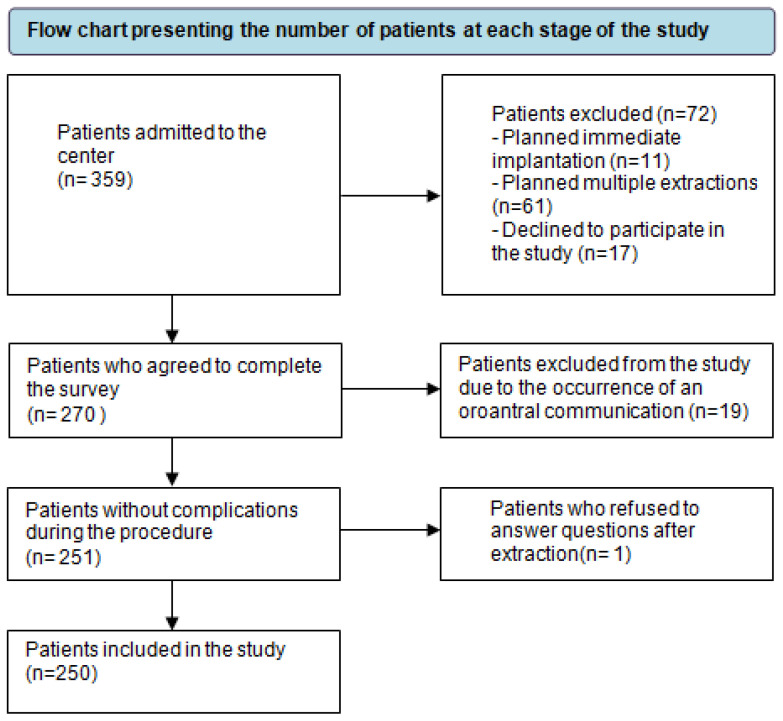
Flow chart of study participants.

A total of 359 patients were assessed for eligibility. Of these, 72 were excluded as they did not meet the eligibility criteria or declined to participate in the study. The remaining 270 patients underwent extraction. Due to the occurrence of an oroantral communication, 19 patients were excluded from the subsequent analysis. Of the 251 patients, one declined to respond to the post-extraction questionnaire, and the responses of the remaining 250 patients were analysed. Figure 2 presents a flow diagram illustrating the inclusion and exclusion of study participants.

### 3.2. Descriptive Data

Table 2 presents the basic demographic and clinical characteristics of the patients studied in both groups. The 250 respondents were distributed as follows: 40% were male and 60% were female. The mean age of the participants was 29.41 years (standard deviation (SD) = 12.82), with the majority of respondents belonging to the age group ≤30 years (66.8%). The mean total score for dental anxiety before the procedure on the Visual Analogue Scale (VAS) was 5.23 (standard deviation (SD) = 2.72). The VAS score was used to categorize the subjects into three groups: those with low anxiety (1–4 VAS score, 39.6%), those with moderate or high anxiety (5–6 VAS score, 22.8%), and those with dental phobia (7–10 VAS score, 37.6%) [22]. The study demonstrated that younger individuals were considerably more prone to seeking extraction of retained teeth than those in older age groups. Additionally, a significantly higher level of preoperative anxiety was observed in patients with retained teeth, whereas postoperative anxiety levels were not significantly different from those observed in the same group of patients. Patients presenting at the clinic for extraction of erupted teeth exhibited significantly higher levels of preoperative pain than the remainder of the patient population.

### 3.3. Main Results

The largest group of respondents (*n* = 143) was assigned to Group A, which exhibited the lowest pre-treatment pain levels. The patients in this group had previously undergone tooth extraction with the intention of retaining the tooth. Group B comprised patients with a history of tooth extraction but with a planned extraction of an erupted tooth. The group of 38 patients exhibited the lowest pre-procedure (4.05) and post-procedure (2.44) stress levels of all the groups, despite having the highest pre-procedure pain levels (3.21). The respondents in this group exhibited the highest mean age (43.53). Group C, which comprised respondents with no history of extraction and a planned extraction of a retained tooth, exhibited the highest level of stress among patients before the procedure (5.97) and after the procedure (4.21). Group D was the smallest of the four groups, with only 12 respondents. The patients in this group had no history of tooth extraction and were scheduled for removal of an erupted tooth. The group exhibited the highest average level of preoperative pain. In contrast, the mean age was lowest among the groups at 22.25 years. In contrast, statistical tests did not demonstrate a statistically significant relationship between the age, gender, anxiety, or bullying level of patients and their group allocation. All these data are presented in Table 3.

Table 4 presents the distribution of respondents across the four age groups proposed by the WHO [23], accompanied by the average levels of anxiety associated with each group. The largest age group of respondents was represented by 114 individuals under the age of 25. The highest level of anxiety was observed in this group (5.56). The lowest levels of anxiety were observed in patients over the age of 60, with an average level of anxiety of 3.4. The results indicate that as age increases, the level of extraction anxiety decreases. Nevertheless, it is important to note that this may be attributed to the fact that younger individuals were more likely to undergo the extraction of retained teeth than older individuals. A statistical analysis revealed a statistically significant correlation between patients’ age and their level of stress (*p* = 0.03). Nevertheless, the Scheffe test did not reveal a significant difference between the individual groups.

Table 5 illustrates the correlation between place of residence and the average level of extraction anxiety. The respondents from rural areas exhibited the lowest level of anxiety (4.82), while those residing in small towns with up to 10,000 residents exhibited the highest level (5.73). The respondents from cities with populations between 100,000 and 1,000,000 exhibited comparable levels of anxiety, with an average score of 5.22. Nevertheless, no statistically significant relationship was found between the parameters in question.

Table 6 presents the results of the average level of anxiety about extraction based onthe level of education. The average levels of anxiety exhibited by respondents from all levels of education were found to be similar. For patients with higher and primary education, the average anxiety level was 5.33, while patients with vocational and secondary education had anxiety levels of 5 and 5.13, respectively. The results of the statistical tests also indicated that there was no significant relationship between the data.

The distribution of stressors affecting respondents differs between groups. Patients with a history of extractions most frequently cited fear of complications of the procedure and pain after extraction, while patients who had never had an extraction most frequently cited complications and pain during the procedure. It should be noted, however, that statistical tests did not demonstrate a statistically significant difference between the datasets. A comprehensive account of the stressors most frequently identified by respondents can be found in Table 7.

The BPCQ scale is divided into three subscales, each representing a distinct area of patients’ beliefs. The lowest anxiety group demonstrated the strongest conviction in their role in pain control, while the highest anxiety group exhibited the strongest conviction in the role of physicians in controlling their pain. The results of each subscale are presented in Table 8. The BPCQ scale is divided into three subscales, each representing a different area of patients’ beliefs. The lowest anxiety group demonstrated the strongest conviction in their role in pain control, while the highest anxiety group exhibited the strongest conviction in the role of physicians in controlling their pain. Nevertheless, statistical tests indicated that there were no significant correlations between stress levels and any of the three groups of the BPCQ scale. Although the result for intrinsic factors was the closest to significance, it did not exceed the *p* < 0.05 threshold. The results of each subscale are presented in Table 8.

The results of the comparison of pain level to preoperative anxiety level based on the level of tooth eruption are shown in Table 9. The results from the table show that as the pain experience increases, the stress level also increases. A statistical analysis also revealed a significant correlation between patients’ stress levels and their pain levels. The results demonstrated that patients exhibiting higher levels of pain also exhibited higher levels of stress prior to the tooth extraction procedure.

## 4. Discussion

### 4.1. Interpretation

Despite the limitations of the study, the results allowed for the drawing of important conclusions. The most significant of these findings is that pain sensations do not represent the most influential factor in patients’ feelings of anxiety. Furthermore, the authors of the study corroborated the findings of other authors who had previously suggested that female gender was a more significant predictor of higher levels of perioperative anxiety [4,24,25,26,27,28,29]. The type of extraction also had a significant effect on the levels of stress experienced by patients. Those undergoing extraction of retained teeth or germectomy were characterised by significantly higher levels of stress, regardless of pain levels. This is demonstrated in Table 9. The results of the BPCQ indicated that patients who exhibited greater confidence in their ability to self-manage pain exhibited lower levels of perioperative anxiety. In such circumstances, it may be beneficial to inform patients of the potential influence they may have on the intensity of discomfort following tooth extraction. Nevertheless, statistical tests did not confirm this. This may be associated with too small a study group. It is noteworthy that patients with a history of tooth extraction were more likely to identify postoperative pain as the primary source of stress. This may be attributed to inadequate adherence to post-extraction instructions or inadequate communication of them. Other authors have also corroborated the assertion that a history of extractions has a significant impact on preoperative anxiety levels [30,31]. It is also possible that patients may exaggerate their recollection of the pain associated with the procedure, as well as afterwards, as the McNeil et al. study demonstrated [32]. The authors of the study demonstrated that anxiety levels were comparable at all levels of patient education and that there were no statistically significant differences. In contrast, Muneer et al. demonstrated that individuals with higher levels of education exhibited significantly higher levels of stress [29]. In contrast, when it came to place of residence, patients from rural areas exhibited the lowest levels of anxiety, while those from small towns with a population of up to 10,000 exhibited the highest levels of anxiety. The results of the study, which were confirmed by statistical tests, also demonstrated that the age of the patient influences the level of stress experienced prior to tooth extraction. The results of the study by Xu and Xia and Gazal et al. demonstrated the opposite of the aforementioned findings, with no evidence of a relationship between age and stress levels prior to tooth extraction [33,34].It is postulated that older individuals are more adept at rationalising their circumstances, which may explain why they are less prone to irrational anxiety [24].

### 4.2. Limitations

Although the study is comprehensive, it is important to be aware of certain limitations when interpreting the results. Firstly, the survey only included patients who had accessed the public health sector at a single centre. The number of operators performing the procedures was limited, which may have influenced the patients’ perceptions. It is possible that their responses were related to the behaviour and skills of the medical staff. A further limitation of the study is the absence of an evaluation of parameters such as respiratory rate and blood pressure, which would allow for correlation with the level of anxiety. In the absence of these data, we are obliged to rely on patients’ opinions, which may not always be reliable or pertinent to the clinical situation. Furthermore, the qualitative survey method does not permit the use of interactive inquiries. Consequently, the findings should be regarded as descriptive and explanatory, rather than as a robust source of theory generation. To the best of the authors’ knowledge, this is the first paper to utilise the BPCQ to investigate peri-extraction anxiety. Consequently, it is not possible to relate the results of this segment of the work to other authors’ research on this topic. Furthermore, another limitation of the study is the relatively limited diversity within the study group, which is a consequence of the single-centre nature of our investigation. Consequently, the generalisability of our findings to broader patient demographics may be somewhat constrained.

### 4.3. Conclusions

The findings by the authors contribute to a more comprehensive understanding of the issue of anxiety in patients undergoing tooth extraction. The results demonstrated that anxiety persists even after extraction is completed, which may result in an inadequate comprehension of post-extraction recommendations. This argument supports the proposition that post-extraction recommendations should be explained with greater accuracy and that they should be communicated in writing in addition to orally. It is of concern that patients with a history of tooth extraction exhibited significantly higher levels of fear of post-extraction pain, which may indicate inadequate adherence to recommendations in previous extractions. For the same reasons, it is also important to consider the potential impact of extraction on patients without a history of the procedure and those undergoing extraction of retained teeth, who demonstrated significantly higher levels of stress than the other groups. To eliminate potential biases in the results, future studies should be conducted on a larger sample of respondents and should be multicentre to increase the demographic diversity of patients surveyed. Moreover, the possibility of extending the survey to include respondents’ past experiences with dentists is worthy of consideration. This could demonstrate whether, for instance, negative experiences with dentists from other specialities are associated with peri-extraction stress. Furthermore, the reliability of responses regarding anxiety or stress should be confirmed by an objective test, such as measuring heart rate before, during, and after the extraction procedure. It is also important to consider additional factors that could be clinically relevant. These include the teeth that were extracted, the duration of the procedure, the type of anaesthesia that was performed before the extraction, and the effect of postoperative wound suturing on patient perceptions.

## Figures and Tables

**Table 1:** The inclusion and exclusion criteria for qualifying patients for the study.

Inclusion Criteria	Exclusion Criteria
Patient consent for the study Performance of tooth extraction or germectomy	1. Immediate implantation after extraction 2. Inability to complete the questionnaire 3. Consent revoked after extraction 4. Multiple extractions during the visit 5. Occurrence of an oroantral communication 6. Patients with psychiatric conditions 7. Unwillingness to complete the survey

**Table 2:** Baseline characteristics of study participants.

	Patients with Fully Erupted Teeth (*n* = 50)	Patients with Impacted Teeth (*n* = 200)	Statistical Significance
Age, years ^a^	38.42(SD = 17.51)	27.16(SD = 10.23)	*p* = 0.001
Sex (%)			*p* = 0.055
Female	24(48%)	126(63%)	
Male	26(52%)	74(37%)	
Level of anxiety ^a^			
Preoperative	4.3	5.44	*p* = 0.007
Postoperative	2.84	3.32	*p* = 0.229
Level of pain before procedure ^a^	3.44	1.57	*p* = 0.001

Factors that may distort the results of the study are the disproportion of patients between the groups and the significant difference in mean age for the two groups. ^a^ Data are presented as mean.

**Table 3:** Baseline characteristics for each group.

	Group A	Group B	Group C	Group D	Statistical Significance
Age, years *	28.33 (SD = 11.05)	43.53 (SD = 16.89)	24.21 (SD = 7.08)	22.25 (SD = 5.63)	*p* = 0.808
Respondents	143	38	57	12	
Male	59	17	15	9	
Female	84	21	42	3	*p* = 0.563
Level of anxiety ^a^					
Preoperative	5.23	4.05	5.97	5.08	*p* = 0.785
Postoperative	2.97	2.44	4.21	4.08	*p* = 0.129
Level of pain before procedure ^a^	1.51	3.21	1.72	4.17	*p* = 0.636

* Data are presented as mean. ^a^ Data are presented as mean.

**Table 4:** Dependence of anxiety on age group.

Age Group	Number of Respondents	Average Level of Anxiety before Procedure	Statistical Significance *p* = 0.031
<25	114	5.56	
25–44	105	5.18	
45–60	21	4.33	
>60	10	3.4	

**Table 5:** Relationship between place of residence and average level of anxiety about extraction.

Residence	Number of Respondents	Average Level of Anxiety Before Procedure	Statistical Significance *p* = 0.733
Rural area	28	4.82	
City of up to 10,000 inhabitants	19	5.73	
City of up to 100,000 inhabitants	94	5.22	
City of more than 100,000 inhabitants	109	5.23	

**Table 6:** Effect of education level on extraction anxiety levels.

Education Level	Number of Respondents	Average Level of Anxiety before Procedure	Statistical Significance *p* = 0.361
Primary	30 (12%)	5.33	
Vocational	18 (7.2%)	5	
Secondary	121 (48.4.5%)	5.13	
Higher	81 (32.4%)	5.33	

**Table 7:** Distribution of the stressors that were most frequently chosen by each group.

Stressor	Group A	Group B	Group C	Group D	Statistical Significance *p* = 0.104
Anaesthesia	20 (14%)	9 (23.7%)	9 (15.8%)	2 (16.7%)	
Pain during removal	32 (22.4%)	5 (13.2%)	17 (29.8%)	5 (41.7%)	
Pain after removal	35 (24.5%)	5 (13.2%)	10 (17.5%)	1 (8.3%)	
Complications	56 (39.2%)	17 (44.7%)	21 (36.8%)	4 (33.3%)	

**Table 8:** Scores of the BPCQ scale based on the level of anxiety.

	BPCQ Subscale		
Levels of Anxiety	Internal Factors	Power of Doctors	Chance Events
1–3	18.01	14.66	12.85
4–6	16.99	14.46	13.24
7–10	17.29	14.94	12.69
Statistical significance	*p* = 0.223	*p* = 0.574	*p* = 0.566

**Table 9:** Influence of pain level on anxiety depending on the level of tooth eruption.

	Average Level of Anxiety before Procedure		Statistical Significance *p* = 0.002
Level of Pain before Procedure	Patients with Erupted Teeth	Patients with Retained Teeth	
1–3	3.41 (*n* = 31)	5.39 (*n* = 182)	
4–6	4.88 (*n* = 9)	5.64 (*n* = 14)	
7–10	6.5 (*n* = 10)	7.00 (*n* = 4)	

## Data Availability

The original contributions presented in the study are included in the article, further inquiries can be directed to the corresponding author.
